# Supplementary material for: Quantum electromechanics on silicon nitride nanomembranes
Source: Nat Commun. 2016 Aug 3;7:12396. doi: 10.1038/ncomms12396 (PMC4976205; doi:10.1038/ncomms12396)
Supplement: Supplementary Information — Supplementary Figures 1-3, Supplementary Notes 1-8 and Supplementary References [file ncomms12396-s1.pdf]

SUPPLEMENTARY FIGURES

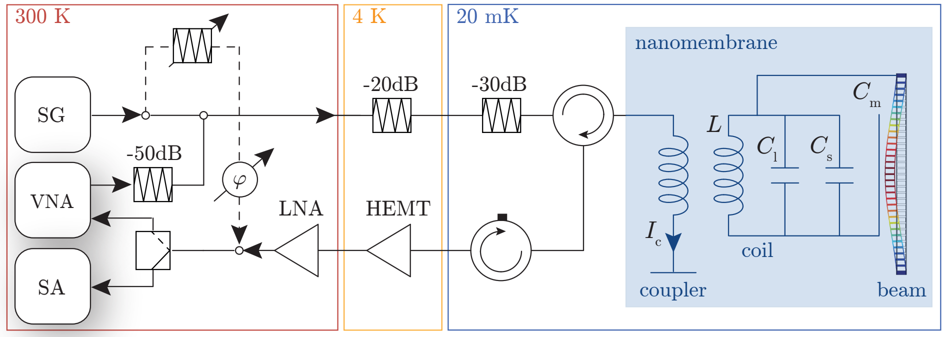

Supplementary Figure 1: **Experimental setup.** The output tone of a microwave signal generator (SG) and the output tone of a vector network analyzer (VNA) are combined at room temperature, attenuated, routed to the sample at about 20 mK and inductively coupled to the LC circuit on the nanomembrane. We detect the reflected output tone after amplification with a high electron mobility transistor amplifier (HEMT), further amplification with a low noise amplifier (LNA), and switchable pump tone cancelation (dashed lines). The measurement is done either phase coherently with the VNA, or we detect the incoherent power spectrum with the spectrum analyzer (SA).

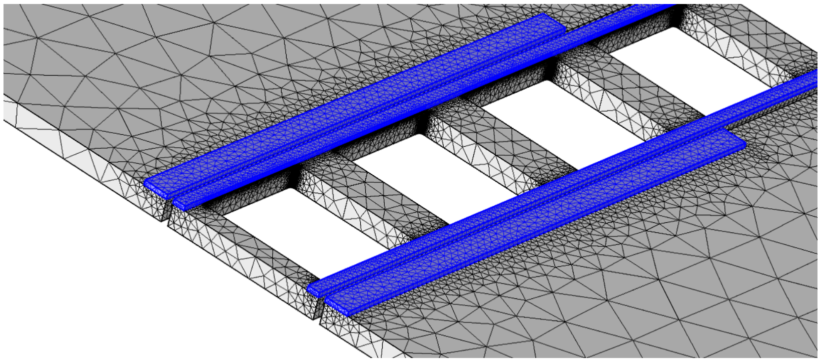

Supplementary Figure 2: **Geometry for high frequency breathing mode simulation.** The simulated structure with 65 nm thick Aluminum (highlighted blue) on 300 nm thick silicon nitride (gray) is shown. We use a symmetric boundary condition in the center of the beam (bottom left).

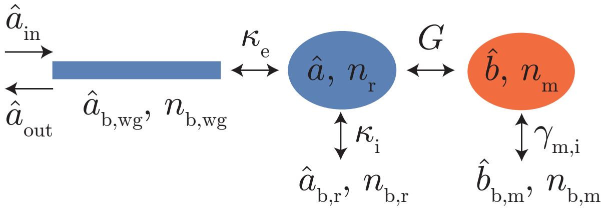

Supplementary Figure 3: **System modes, coupling rates and noise baths.** In the reflective geometry the microwave cavity mode $\hat{a}$ is coupled to the coherent waveguide modes $\hat{a}_{\rm in}$ and $\hat{a}_{\rm out}$ with the external coupling strength κ_e. It is also coupled to a bath of noise photons, ideally at the refrigerator temperature $n_{b,r}$, with the intrinsic coupling strength κ_i. In addition, the waveguide bath mode $\hat{a}_{b,\rm wg}$ can be populated with thermal noise photons $n_{b,\rm wg}$, which also couples with κ_e. The mechanical resonator mode $\hat{b}$ is coupled to the microwave resonator with the parametrically enhanced electromechanical coupling strength G. In addition, it is coupled to a bath of noise phonons, ideally at the refrigerator temperature $n_{b,m}$, with the intrinsic coupling rate $\gamma_{m,i}$.

SUPPLEMENTARY NOTES

Supplementary Note 1: Circuit properties

Coil simulation

Our device is fabricated and simulated on a 300 nm thick and (777 μm)^2 large Si_3N_4 membrane. The coil wire is 500 nm wide and 120 nm thick, with a 1 μm pitch, 42 turns forming a square with lateral length of only 87 μm, well in the lumped element limit. According to finite element simulations, which includes wire cross-overs, the coil is inductive up to its self resonance frequency of $\nu_{\rm srf} = 13.38$ GHz, where the half wavelength roughly matches the total wire length of $l = 7.7$ mm. We repeat this simulation with a small additional shunt capacitor of known value ($\Delta C = 0.1$ fF) and extract the new self resonance frequency $\nu_{\rm srf,2}$. Solving the two simple relations $\omega_{\rm srf} = (LC_{\rm l})^{-1/2}$ and $\omega_{\rm srf,2} = (L(C_{\rm l} + \Delta C))^{-1/2}$, we extract $L = 68$ nH and $C_{\rm l} = 2.1$ fF. These results are valid close to - but below - the self resonance frequency of the coil. In this limit we realize a maximum impedance of $Z_0 = \sqrt{L/C_{\rm l}} \approx 5.7$ kΩ, far exceeding the vacuum impedance $Z_{\rm vac} \approx 377$ Ω, and approaching the resistance quantum $R_{\rm q} = h/(2e)^2 \approx 6$ kΩ.

Full circuit parameters

Knowing the inductance L of the fabricated inductor, as well as the actually measured resonance frequency of $\omega_{\rm r}/(2\pi) = 7.965$ GHz, yields a total capacitance of $C_{\rm tot} = C_{\rm l} + C_{\rm m} + C_{\rm s} = 5.9$ fF and a total circuit impedance of $Z_{\rm tot} = 3.4$ kΩ (see Supplementary Figure 1). The modulated capacitance $C_{\rm m}$ is a function of the capacitor slot size, which we estimate from numerical simulations of the measured coupling strength to be 62 nm. Numerical finite element simulations yield a nanobeam capacitance of $C_{\rm m} \approx 1.6$ fF for this gap size (see Figs. in main text), which gives a participation factor of $\eta \approx 0.27$.

Using a self resonance frequency simulation of the full electrical circuit including $C_{\rm m}$, we can attribute the remaining stray capacitance of $C_{\rm s} \approx 2.2$ fF to the coil to capacitor wiring (62 %), the presence of a second resonant circuit (15 %), the coupling wire (8 %), non-ideal crossovers (8 %). The remaining 0.15 fF (7 %) we attribute to frequency dependence, packaging and our uncertainty of the relative permittivity of silicon nitride at low temperature $\epsilon_{\rm r} \approx 7.5-8$. As expected, for these full circuit simulations we extract the same inductance L as for the coil only simulations. The value of $L = 68$ nH is consistent with both, the modified Wheeler and the current sheet method [1], to within ± 2 nH.

High frequency mechanical mode

In order to estimate the electromechanical coupling of the high frequency acoustic mode, we consider that the identical microwave circuit is coupled to both sides of the nanobeam. Here the outer capacitor length is taken to match the acoustic defect region of 2×3 lattice constants, see Supplementary Figure 2. We find a reduced participation ratio $\eta = 0.14$ due to the reduced $C_{\rm m} \approx 0.7$ fF in this case. Further improvements in reducing the circuit's stray capacitance will have a big impact for efficient coupling to high frequency modes.

Supplementary Note 2: Device Fabrication

Wafer preparation

After a thorough RCA clean (organic and particle clean, oxide strip, ionic clean) [2], we grow a 300 nm thick film of stoichiometric Si_3N_4 at a temperature of 835 C, using low pressure chemical vapor deposition on both sides of a doubly polished 200 μm thick, high resistivity (>10 kΩ-cm), Si $\langle 100 \rangle$ wafer. After cooldown, the dielectric film has a stress of ≈ 1 GPa due to the differential expansion coefficient. We spin a protective layer of photoresist and dice the wafer in 10 mm $\times$10 mm chips.

Membrane patterning

The chips are cleaned using weak sonication in acetone (ACE) and isopropyl alcohol (IPA) and prebaked at 180 °C for 2 min on a hotplate. We then spin the front side with ZEP 520A at 67 Hz (4000 rpm) for protection, bake at 180 °C for 2 min, spin the back side with ZEP 520A at 33 Hz (2000 rpm) and bake at 180 °C for 2 min. Patterning of the 16 membrane areas of size 1 mm ×1 mm each, is done with 100 keV electron beam exposure with a 200 nA electron beam, 50 nm fracturing size and a dose of 250 μC/cm^2 on the chip back side. This layer is carefully aligned to the chip corners. We develop with ZND-N50 for 2.5 min and rinse in methyl isobutyl ketone (MIBK) for 0.5 min. This is followed by an ICP-RIE etch of the silicon nitride in the developed areas, using a C_4F_8 (0.57 cm^3/s = 34 sccm) / SF_6 (0.2 cm^3/s = 12 sccm) plasma, generated with an ICP power of 1000 W, RF power of 30 W and a DC bias of 84 V, at a pressure of 2000 Pa (15 Torr) and a temperature of 25 °C for 7 min 15 s. We finish this layer by a thorough cleaning of the chips using weak sonication in TCE, IPA, ZDMAC, ACE and IPA.

Nanobeam patterning and membrane pre-etching

This layer initially follows the same procedure to pattern the top side of the chip (no resist on the back side) with the nanobeams, pull-in cuts and the global and pattern alignment markers of size (20 μm)^2, with these process parameters: 300 pA beam, 2.5 nm fracturing, 275 μC/cm^2 dose, 7 min 50 s etch time. We then use an o-ring sealed holder to expose only the back side of the chip to 30 % KOH in water at 85 °C with the stir bar at 7 Hz (400 rpm). This anisotropic Si wet etch is stopped when the wafer becomes semi-transparent (dark orange) in the membrane area, when illuminated with an LED on the sealed side of the chip. The color indicates a silicon thickness of ≈ 5 μm which is usually achieved after 2.5 h of etching. After cleaning the chip in ultra-pure deionized water and IPA, we wet etch both the front and back side of the chip in 30 % KOH in water at 65 °C with the stir bar at 1.7 Hz (100 rpm) for 70 s. This partially undercuts ($\approx$ 100 nm) the nanobeams for a clean subsequent inverse shadow evaporation process [3], used to pattern the small gapped capacitors. The chips are then rinsed in hot water, fresh piranha solution (mix 45 mL H_2SO_4 with 15 mL H_2O_2 at 85 °C with the stir bar at 5 Hz, 300 rpm) for 8 min followed by a water and IPA rinse.

Capacitors and ground plane

This layer patterns all of the electrical circuit, except for the coil wires. We start with a prebake at 180 °C for 2 min, and spin the front side with ZEP 520A at 33 Hz (2000 rpm), followed by another bake at 180 °C for 2 min. We use 100 keV electron beam lithography to pattern the ground plane and transmission lines (200 nA beam, 50 nm fracturing, 290 μC/cm^2 dose with PEC), as well as the capacitor wires, and the wires connecting the capacitors with the coil end and center (10 nA beam, 10 nm fracturing, 275 μC/cm^2 dose). This layer is carefully aligned to the etched negative markers from the previous step. We develop the chips in the same way and use a O_2 plasma ash process (0.83 cm^3/s = 50 sccm O_2, 0.74 bar, 13.56 MHz, 35 W, 2 min) to descum the surface before deposition of aluminum. For the deposition we use an electron beam evaporator (0.3 nm/s, 65 nm thickness at $1 \cdot 10^{-5}$ Pa to $2 \cdot 10^{-5}$ Pa). We then do a lift-off process in 80 °C NMP for > 1 h and carefully rinse in ACE and IPA.

Scaffolding layer

Now we pattern a scaffolding layer to fabricate the cross-overs. After prebaking, we spin LOR 5B at 50 Hz (3000 rpm) and bake at 180 °C for 5 min, followed by spinning PMMA 950k A2 at 67 Hz (4000 rpm) and baking at 180 °C for 5 min. We then beam write the negative pattern of the cross-over support structure using aligned electron beam lithography (200 nA beam, 25 nm fracturing, 1000 μC/cm^2 dose). The resist is developed using MIBK:IPA (1:3) for 1 min, and rinsed in IPA for 30 s. We then wet etch the scaffolding layer using MF-319 for 8 s, followed by a water rinse and IPA which stops the etch. Finally we remove the remaining PMMA layer with ACE (30 s) and reflow the LOR cross-over support layer on a hot plate at 200 °C for 10 min. This creates a structurally stable arc shaped cross over scaffolding.

Coil wire patterning

In order to pattern the narrow pitch coils, we spin PMMA 495 A8 at 33 Hz (2000 rpm), bake, spin PMMA 950k A2 at 33 Hz (2000 rpm) and bake again. Then we lithographically define the coil wires, which overlap the capacitor wires (10 nA beam, 10 nm fracturing, 1800 μC/cm^2 dose) and develop the resist as described previously. Development is followed by the same plasma ashing, deposition of aluminum (1 nm/s, 120 nm, p$\approx 2 \cdot 10^{-5}$ Pa) and lift off, during which the NMP (at 80 °C, 3 h) dissolves the LOR scaffolding layer.

DC contact wire

After a careful rinse with ACE and IPA we reproduce the previous layer recipe to pattern a small (500 nm$\times$4 μm) DC contact wire that symmetrically covers all overlap regions between capacitor wire and coil wire (two per coil and capacitor). Here we use an in-situ ion gun etch process (normal incidence with 4 cm gridded Kaufman source, 400 V, 21 mA for 5 min) right before the aluminum deposition of thickness 140 nm, in order to establish reliable contact. Contact is tested after lift-off on DC test structures of the same contact size located in the center of the chip. High resistance contacts with low capacitance at microwave frequencies would lead to additional parasitic in-series capacitances of the fabricated circuit.

Release

For the final release step we prepare a silicon enriched solution of TMAH to selectively etch the silicon without aluminum corrosion [4, 5]. We use a custom built reactor vessel with thermometer port and a hotplate with magnetic stir bar to mix 60 g of TMAH (25 %, 6N) and 250 g water, and then add 5.12 g of silicon powder (-325 mesh, 5N) and stir at 5 Hz (300 rpm). After the chemical reaction calms down we start heating the solution up to 80 °C. When the solution is clear, we wait for 1 h and prepare a clear mixture of 5.21 g of TMAH (25 %, 6N) and 2.11 g of the oxidizing agent ammonium persulfate in a small beaker. We add the mixture to the solution with the stir bar at 17 Hz (1000 rpm), wait 10 min to 15 min, reduce the stir speed and add the sample in a vertical position. The sample is securely clamped, but with the membranes open to a steady flow of solution on the back and front side of the chip. We keep the solution at 80 °C and wait for the membranes to become fully transparent (1 h to 2 h). As a last step we carefully remove the sample, rinse it throughly in hot water, cold water, IPA, ultra purified IPA, and dry it using a CO_2 critical point dryer.

Supplementary Note 3: Experimental setup

For the measurements of coherent and incoherent electromechanical response, we combine the output of a vector network analyzer with up to two microwave sources, feed the microwave tones to the base plate of a cryogen free dilution refrigerator using UT-085 stainless steel coaxial cables with feedthroughs for thermalization at each temperature stage and an additional attenuation of 50 dB to suppress room temperature Johnson noise (see Supplementary Figure 1). We couple to the sample in a reflective geometry using a circulator and a low loss, high dielectric constant, copper printed circuit board (PCB). On the PCB and chip we use 50 Ω coplanar waveguides to route the microwave tones all the way to the membrane with very little reflections. On the membrane the center conductor is shorted to ground through a small wire, which couples the waveguide inductively to the LC resonant circuit.

On the output side we use another circulator for isolating the sample from 4 K noise, otherwise entering in reverse direction. Crimped niobium titanium superconducting cables are used to connect directly to a low noise, high electron mobility transistor amplifier (HEMT) at 4 K. From there we use lowloss UT-085 stainless steel - beryllium copper cables and amplify once more at room temperature. In order to suppress spurious response peaks in high drive power cooling measurements, we add a phase and amplitude adjusted part of the pump tone to the output signal, as shown in Supplementary Figure 1. Using a phase shifter we compensate for about 3 m of path length difference and typically achieve a carrier suppression of > 40 dB.

Supplementary Note 4: Derivation of cavity response functions

Fourier Transform

We use the following convention for the Fourier transform. Given an operator $\hat{A}$ we define

$$\hat{A}(t) = \frac{1}{\sqrt{2\pi}} \int_{-\infty}^{+\infty} d\omega e^{-i\omega t} \hat{A}(\omega) \tag{1}$$

$$\hat{A}(\omega) = \frac{1}{\sqrt{2\pi}} \int_{-\infty}^{+\infty} dt e^{i\omega t} \hat{A}(t). \tag{2}$$

Reflective coupling to a microwave resonator

We consider a resonator mode $\hat{a}$ at frequency ω_r, which is coupled to a single waveguide with coupling strength κ_e, and to the environment with the coupling strength κ_i (see Supplementary Figure 3). We follow general input-output theory [6] to write the time derivative of the annihilation operator

$$\dot{\hat{a}}(t) = -\left(i\omega_r + \frac{\kappa}{2}\right)\hat{a}(t) - \sqrt{\kappa_e}\hat{a}_{\text{in}}(t) - \sqrt{\kappa_i}\hat{a}_{b,r}(t) - \sqrt{\kappa_e}\hat{a}_{b,\text{wg}}(t), \tag{3}$$

where $\kappa = \kappa_e + \kappa_i$ is the total resonator linewidth, $\hat{a}_{\text{in}}(t)$ represents the annihilation operator of the coherent input mode, $\hat{a}_{b,\text{wg}}(t)$ the waveguide mode operator, and $\hat{a}_{b,r}(t)$ is the respective field operator of the resonator environment. We take the Fourier transform to remove the time derivative, and simplify to get the frequency dependence

$$\hat{a}(\omega) = \frac{-\sqrt{\kappa_e}\hat{a}_{\text{in}}(\omega) - \sqrt{\kappa_i}\hat{a}_{b,r}(\omega) - \sqrt{\kappa_e}\hat{a}_{b,\text{wg}}(t)}{\kappa/2 + i(\omega_r - \omega)}. \tag{4}$$

The resonator output field is defined as

$$\begin{aligned}\hat{a}_{\text{out}}(\omega) &= \hat{a}_{\text{in}}(\omega) + \sqrt{\kappa_e}\hat{a}(\omega) \\ &= \hat{a}_{\text{in}}(\omega) + \frac{-\kappa_e\hat{a}_{\text{in}}(\omega) - \sqrt{\kappa_e\kappa_i}\hat{a}_{b,r}(\omega) - \kappa_e\hat{a}_{b,\text{wg}}(t)}{\kappa/2 + i(\omega_r - \omega)},\end{aligned} \tag{5}$$

which we can use to calculate the complex scattering parameter as measured by a network analyzer

$$S_{11}(\omega) = \frac{\langle\hat{a}_{\text{out}}(\omega)\rangle}{\langle\hat{a}_{\text{in}}(\omega)\rangle} = 1 - \frac{\kappa_e}{\kappa/2 + i(\omega_r - \omega)}, \tag{6}$$

where the incoherent bath mode terms drop out. We use this function to simultaneously fit the real and imaginary part of the measured cavity response and extract the intrinsic and extrinsic cavity coupling rates.

Drive photon number

It is useful to define the intra-cavity photon number n_d due to a classical coherent drive tone at frequency ω_d. We replace the field operators in Supplementary Equation 4 with the classical amplitudes $\hat{a}(\omega) \to \alpha(\omega)$ and discard the resonator and waveguide bath modes to get

$$n_d = |\alpha_{\text{d}}|^2 = |\alpha_{\text{in}}|^2 \frac{4\kappa_e}{\kappa^2 + 4\Delta_{r,d}^2}. \tag{7}$$

Here we have introduced the resonator drive detuning $\Delta_{r,d} = \omega_r - \omega_d$ and the input photon flux $|\alpha_{\text{in}}|^2 = P_{\text{in}}/(\hbar\omega_d)$. The power at the cavity input can be expressed as $P_{\text{in}} = 10^{-3}10^{(\mathcal{A}+P_{\text{d}})/10}$ with P_{d} the drive power in dBm and $\mathcal{A}$ the total attenuation of the input line in dB.

Asymmetric lineshape

Fano line shapes generally originate from interference between a resonant mode and a background mode [7]. Experimental imperfections, such as leakage or reflections in the feedline circuit, can lead to such asymmetric cavity line shapes. We can model this effect by introducing a complex valued external resonator to waveguide coupling parameter $\bar{\kappa}_{\rm e} = |\kappa_e| e^{-iq}$, where q is a version of the Fano parameter. While small q values do not change the magnitude of the inferred external coupling (or the drive photon number), they correctly model small asymmetries in the Lorentzian cavity response [8]. For simplicity we define the generalized coupling $\bar{\kappa}_{\rm e} = \kappa_e - iq$ and substitute into Supplementary Equation 6, to get the generalized resonator line shape

$$S_{11}(\omega) = 1 - \frac{\kappa_e - iq}{\kappa/2 - i(\omega_r - \omega)}. \tag{8}$$

Supplementary Note 5: Derivation of cavity electromechanical response functions

In this section we follow previous work [9–12] to calculate the coherent response and the full noise spectrum of the system. In contrast to earlier treatments we also include thermal noise in the feedline circuit, which gives rise to an increased mechanical occupation and an asymmetric cavity noise line shape.

System Hamiltonian and equations of motion

The Hamiltonian of the coupled microwave cavity-mechanical system (see Supplementary Figure 3) can be written as

$$\hat{H} = \hbar\omega_r \hat{a}^\dagger \hat{a} + \hbar\omega_m \hat{b}^\dagger \hat{b} + \hbar g_0 \hat{a}^\dagger \hat{a}(\hat{b}^\dagger + \hat{b}), \tag{9}$$

where $\hat{b}$ ($\hat{b}^\dagger$) is the annihilation (creation) operator of the mechanical mode at frequency ω_m, and g_0 is the electromechanical coupling strength, i.e. the resonator frequency shift due to a mechanical displacement corresponding to half a phonon on average. We excite the microwave resonator mode using a strong drive tone at frequency $\omega_{\rm d}$, detuned from the resonator frequency by $\Delta_{r,d} = \omega_r - \omega_d$. The linearized Hamiltonian in the rotating frame is then given as

$$\hat{H}' = -\hbar\Delta_{r,d} \hat{a}^\dagger \hat{a} + \hbar\omega_m \hat{b}^\dagger \hat{b} + \hbar G(\hat{a}^\dagger + \hat{a})(\hat{b}^\dagger + \hat{b}), \tag{10}$$

where $G = \sqrt{n_{\rm d}} g_0$ is the parametrically enhanced optomechanical coupling strength. The linearized Langevin equations are given as

$$\dot{\hat{a}}(t) = -\left(i\Delta_{r,d} + \frac{\kappa}{2}\right)\hat{a}(t) - iG(\hat{b}(t) + \hat{b}^\dagger(t)) - \sqrt{\kappa_e}\hat{a}_{\rm in}(t) - \sqrt{\kappa_i}\hat{a}_{b,r}(t) - \sqrt{\kappa_e}\hat{a}_{b,{\rm wg}}(t) \tag{11}$$

$$\dot{\hat{b}}(t) = -\left(i\omega_m + \frac{\gamma_{m,i}}{2}\right) - iG(\hat{a}^\dagger(t) + \hat{a}(t)) - \sqrt{\gamma_{m,i}}\hat{b}_{b,m}(t). \tag{12}$$

Taking the Fourier transform and simplifying we obtain

$$\chi_{\rm r}^{-1}(\omega)\hat{a}(\omega) = -iG(\hat{b}(\omega) + \hat{b}^\dagger(\omega)) - \sqrt{\kappa_e}\hat{a}_{\rm in}(\omega) - \sqrt{\kappa_{\rm i}}\hat{a}_{b,r}(\omega) \tag{13}$$

$$\widetilde{\chi}_{\rm r}^{-1}(\omega)\hat{a}^\dagger(\omega) = iG(\hat{b}(\omega) + \hat{b}^\dagger(\omega)) - \sqrt{\kappa_e}\hat{a}_{\rm in}^\dagger(\omega) - \sqrt{\kappa_{\rm i}}\hat{a}_{b,r}^\dagger(\omega) \tag{14}$$

$$\chi_{\rm m}^{-1}(\omega)\hat{b}(\omega) = -iG(\hat{a}(\omega) + \hat{a}^\dagger(\omega)) - \sqrt{\gamma_{m,i}}\hat{b}_{b,m}(\omega) \tag{15}$$

$$\widetilde{\chi}_{\rm m}^{-1}(\omega)\hat{b}^\dagger(\omega) = iG(\hat{a}(\omega) + \hat{a}^\dagger(\omega)) - \sqrt{\gamma_{m,i}}\hat{b}_{b,m}^\dagger(\omega), \tag{16}$$

where we have introduced the uncoupled susceptibilities of the cavity and the mechanical mode

$$\chi_{\rm r}^{-1}(\omega) = \kappa/2 + i(\Delta_{r,d} - \omega) \tag{17}$$

$$\widetilde{\chi}_{\rm r}^{-1}(\omega) = \kappa/2 - i(\Delta_{r,d} + \omega) \tag{18}$$

$$\chi_{\rm m}^{-1}(\omega) = \gamma_{m,i}/2 + i(\omega_m - \omega) \tag{19}$$

$$\widetilde{\chi}_{\rm m}^{-1}(\omega) = \gamma_{m,i}/2 - i(\omega_m + \omega). \tag{20}$$

In the sideband resolved limit $\omega_m \gg \kappa, G$, and for positive detuning of the drive tone $\Delta_{r,d} \approx \omega_m$ (red side pumping), the linearized Langevin equations can be written approximately as,

$$\hat{a}(\omega) = \frac{iG\chi_\mathrm{m}\chi_\mathrm{r}\sqrt{\gamma_{m,i}}\hat{b}_{b,m}(\omega) - \chi_\mathrm{r}(\sqrt{\kappa_\mathrm{e}}\hat{a}_\mathrm{in}(\omega) + \sqrt{\kappa_\mathrm{i}}\hat{a}_{b,r}(\omega) + \sqrt{\kappa_\mathrm{e}}\hat{a}_{b,\mathrm{wg}}(\omega))}{1 + G^2\chi_\mathrm{m}\chi_\mathrm{r}} \tag{21}$$

$$\hat{b}(\omega) = \frac{-\chi_\mathrm{m}\sqrt{\gamma_{m,i}}\hat{b}_{b,m}(\omega) - iG\chi_\mathrm{m}\chi_\mathrm{r}(\sqrt{\kappa_\mathrm{e}}\hat{a}_\mathrm{in}(\omega) + \sqrt{\kappa_\mathrm{i}}\hat{a}_{b,r}(\omega) + \sqrt{\kappa_\mathrm{e}}\hat{a}_{b,\mathrm{wg}}(\omega))}{1 + G^2\chi_\mathrm{m}\chi_\mathrm{r}}. \tag{22}$$

Now we can calculate the cavity output mode

$$\begin{aligned} \hat{a}_\mathrm{out}(\omega) &= \hat{a}_\mathrm{in}(\omega) + \sqrt{\kappa_\mathrm{e}}\hat{a}(\omega) \\ &= \hat{a}_\mathrm{in}(\omega) - (\hat{a}_\mathrm{in}(\omega) + \hat{a}_{b,\mathrm{wg}}(\omega))\frac{\kappa_\mathrm{e}\chi_\mathrm{r}}{1 + G^2\chi_\mathrm{m}\chi_\mathrm{r}} - \hat{a}_{b,r}(\omega)\frac{\sqrt{\kappa_\mathrm{e}\kappa_\mathrm{i}}\chi_\mathrm{r}}{1 + G^2\chi_\mathrm{m}\chi_\mathrm{r}} + \hat{b}_{b,m}(\omega)\frac{iG\sqrt{\kappa_\mathrm{e}\gamma_{m,i}}\chi_\mathrm{m}\chi_\mathrm{r}}{1 + G^2\chi_\mathrm{m}\chi_\mathrm{r}}. \end{aligned} \tag{23}$$

Electromagnetically Induced Transparency

We first calculate the coherent part of the system response using Supplementary Equation 23 and drop incoherent noise terms to get

$$S_{11}(\omega) = \frac{\langle \hat{a}_\mathrm{out}(\omega)\rangle}{\langle \hat{a}_\mathrm{in}(\omega)\rangle} = 1 - \frac{\kappa_\mathrm{e}\chi_\mathrm{r}}{1 + G^2\chi_\mathrm{m}\chi_\mathrm{r}}. \tag{24}$$

Substituting the bare response of the cavity and the mechanical oscillator we get the coherent EIT response function valid for small probe drive strengths

$$S_{11}(\omega) = 1 - \frac{\kappa_\mathrm{e}}{\kappa/2 + i(\Delta_{r,d} - \omega) + \frac{G^2}{\gamma_{m,i}/2 + i(\omega_m - \omega)}}. \tag{25}$$

In order to take into account potential interference with a continuum of parasitic modes, we can follow the procedure outlined above. Substituting $\kappa_\mathrm{e} \to \kappa_\mathrm{e} - iq$ we get

$$S_{11,\mathrm{as}}(\omega) = 1 - \frac{\kappa_\mathrm{e} - iq}{\kappa/2 + i(\Delta_{r,d} - \omega) + \frac{G^2}{\gamma_{m,i}/2 + i(\omega_m - \omega)}}, \tag{26}$$

which can be used to fit asymmetric EIT spectra.

Supplementary Note 6: Quantum derivation of observed noise spectra

Using the Fourier transforms defined above, we can write the spectral density of an operator $\hat{A}$ as

$$S_{AA}(t) = \int_{-\infty}^{+\infty} d\tau e^{i\omega\tau} \left\langle \hat{A}^\dagger(t+\tau)\hat{A}(t)\right\rangle \tag{27}$$

$$S_{AA}(\omega) = \int_{-\infty}^{+\infty} d\omega' \left\langle \hat{A}^\dagger(\omega)\hat{A}(\omega')\right\rangle. \tag{28}$$

The auto-correlation function of the detected normalized field amplitude (or the photo current) of the output mode $\hat{I}(t) = \hat{a}_\mathrm{out}(t) + \hat{a}^\dagger_\mathrm{out}(t)$ is then given as

$$S_{II} = \int_{-\infty}^{+\infty} d\omega' \left\langle \left(\hat{a}_\mathrm{out}(\omega) + \hat{a}^\dagger_\mathrm{out}(\omega)\right)\left(\hat{a}_\mathrm{out}(\omega') + \hat{a}^\dagger_\mathrm{out}(\omega')\right)\right\rangle. \tag{29}$$

Substituting $\hat{a}_\mathrm{out}(\omega)$ and $\hat{a}^\dagger_\mathrm{out}(\omega)$ from Supplementary Equation 23 we find a general expression for the single sided noise spectrum

$$\begin{aligned} S(\omega) = n_{b,\mathrm{wg}}\Big|\Big(1 - \frac{\kappa_\mathrm{e}\widetilde{\chi}_\mathrm{r}}{1 + G^2\widetilde{\chi}_\mathrm{m}\widetilde{\chi}_\mathrm{r}}\Big)\Big|^2 + n_{b,r}\frac{|\kappa_\mathrm{e}\kappa_\mathrm{i}\widetilde{\chi}_\mathrm{r}|^2}{|1 + G^2\widetilde{\chi}_\mathrm{m}\widetilde{\chi}_\mathrm{r}|^2} + n_{b,m}\frac{\kappa_\mathrm{e}\gamma_{m,i}G^2|\widetilde{\chi}_\mathrm{m}|^2|\widetilde{\chi}_\mathrm{r}|^2}{|1 + G^2\widetilde{\chi}_\mathrm{m}\widetilde{\chi}_\mathrm{r}|^2} \\ + (n_{b,\mathrm{wg}} + 1)\Big|\Big(1 - \frac{\kappa_\mathrm{e}\chi_\mathrm{r}}{1 + G^2\chi_\mathrm{m}\chi_\mathrm{r}}\Big)\Big|^2 + (n_{b,r} + 1)\frac{\kappa_\mathrm{e}\kappa_\mathrm{i}|\chi_\mathrm{r}|^2}{|1 + G^2\chi_\mathrm{m}\chi_\mathrm{r}|^2} + (n_{b,m} + 1)\frac{\kappa_\mathrm{e}\gamma_{m,i}G^2|\chi_\mathrm{m}|^2|\chi_\mathrm{r}|^2}{|1 + G^2\chi_\mathrm{m}\chi_\mathrm{r}|^2}. \end{aligned} \tag{30}$$

Here, $n_{b,\mathrm{wg}}$ and $n_{b,r}$ represent the bath of noise photons from the waveguide and the microwave resonator environment respectively; $n_{b,m}$ corresponds to the phonon bath at the mechanical frequency (see Supplementary Figure 3). We assume thermal input noise correlations for all input noise terms, i.e. $\langle \hat{b}_{b,m}(\omega)\hat{b}^{\dagger}_{b,m}(\omega')\rangle = (n_{b,m}+1)\delta(\omega+\omega')$, $\langle \hat{b}^{\dagger}_{b,m}(\omega)\hat{b}_{b,m}(\omega')\rangle = n_{b,m}\delta(\omega+\omega')$, $\langle \hat{a}_{b,r}(\omega)\hat{a}^{\dagger}_{b,r}(\omega')\rangle = (n_{b,r}+1)\delta(\omega+\omega')$, $\langle \hat{a}^{\dagger}_{b,r}(\omega)\hat{a}_{b,r}(\omega')\rangle = n_{b,r}\delta(\omega+\omega')$, $\langle \hat{a}_{b,\mathrm{wg}}(\omega)\hat{a}^{\dagger}_{b,\mathrm{wg}}(\omega')\rangle = (n_{b,\mathrm{wg}}+1)\delta(\omega+\omega')$ and $\langle \hat{a}^{\dagger}_{b,\mathrm{wg}}(\omega)\hat{a}_{b,\mathrm{wg}}(\omega')\rangle = n_{b,\mathrm{wg}}\delta(\omega+\omega')$.

In the sideband resolved regime and positive detuning (red sideband pump) we can drop the terms proportional to $\widetilde{\chi}_\mathrm{m}$ and $\widetilde{\chi}_\mathrm{r}$. In order to represent a realistic experimental setup, we introduce the fixed gain $\mathcal{G}$ in units of dB and the system noise temperature n_add in units of resonator quanta and referenced to the cavity output. We can now write the full expression for the single sided power spectral density as measured by a spectrum analyzer, valid in the presence of all relevant noise baths

$$S(\omega) = \hbar\omega_d 10^{\mathcal{G}/10}\Big[n_\mathrm{add} + n_{b,\mathrm{wg}} + (n_{b,\mathrm{wg}}+1)\Big|\Big(1 - \frac{\kappa_e\chi_\mathrm{r}}{1+G^2\chi_\mathrm{m}\chi_\mathrm{r}}\Big)\Big|^2 + (n_{b,r}+1)\frac{\kappa_e\kappa_\mathrm{i}|\chi_\mathrm{r}|^2}{|1+G^2\chi_\mathrm{m}\chi_\mathrm{r}|^2} + (n_{b,m}+1)\frac{\kappa_e\gamma_{m,i}G^2|\chi_\mathrm{m}|^2|\chi_\mathrm{r}|^2}{|1+G^2\chi_\mathrm{m}\chi_\mathrm{r}|^2}\Big]. \tag{31}$$

We minimize the number of free parameters by eliminating the resonator bath $n_{b,r}$ using the relation

$$\kappa n_\mathrm{r} = \kappa_\mathrm{e} n_{b,\mathrm{wg}} + \kappa_\mathrm{i} n_{b,r}. \tag{32}$$

With the Supplementary Equations 21 and 22 we can calculate [10] the mechanical occupation n_m

$$n_m = n_{b,m}\left(\frac{\gamma_{m,i}}{\kappa}\frac{4G^2+\kappa^2}{4G^2+\kappa\gamma_{m,i}}\right) + n_r\left(\frac{4G^2}{4G^2+\kappa\gamma_{m,i}}\right), \tag{33}$$

which we use to also replace the mechanical bath occupation $n_{b,m}$ in Supplementary Equation 31.

Thermal waveguide noise

At large drive photon numbers we observe a power dependent increase of the measured noise background. Using a cavity filter to remove broad band phase noise of the microwave source did not remove this background. Similarly, the power levels of the observed microwave signals are believed to be far from saturating the HEMT amplifier. However, we found that the noise figure of the low noise amplifier (LNA) is degrading at higher pump powers. This effect explains the observed noise measurement background rise, which we therefore absorb into a small increase of n_add. Based on the implemented filtering of Johnson noise on the input (attenuators) and output lines (circulators / isolators) we estimate the broad band black body radiation entering the chip waveguide to be $n_{b,\mathrm{wg}} \leq 0.01$. See for example Ref. [13] for an independent cavity temperature measurement in a similar setup with approximately 10 dB less attenuation.

Asymmetric noise spectra

In our measurements the cavity noise bath exceeds the waveguide noise bath for all relevant pump powers. In this case the microwave resonator bath n_r manifests itself as a broad band resonator noise peak on top of the background. This power dependent noise peak shows a small asymmetry for the highest pump powers. We follow a similar procedure as outlined above and introduce a complex waveguide coupling constant to find better agreement with the measured data in this limit. We make the substitution $\kappa_\mathrm{e} \to \kappa_\mathrm{e} - iq$ in the first term proportional to $n_{b,\mathrm{wg}}$ in Supplementary Equation 31 and expand it. For $\kappa_e^2 \gg q^2$ we can only keep q to linear order and simplify the expressions. We can then write the asymmetric noise power spectrum with two additional terms as

$$S_\mathrm{as}(\omega) = S(\omega) + \hbar\omega_d 10^{\mathcal{G}/10}(n_{b,\mathrm{wg}}+1)\Big[\frac{2qG^2(\omega_m-\omega)|\chi_\mathrm{m}|^2|\chi_\mathrm{r}|^2}{|1+G^2\chi_\mathrm{m}\chi_\mathrm{r}|^2} + \frac{2q(\Delta_{r,d}-\omega)|\chi_\mathrm{r}|^2}{|1+G^2\chi_\mathrm{m}\chi_\mathrm{r}|^2}\Big]. \tag{34}$$

The additional two terms are odd functions with a vanishing integral. This ensures the same fit results as obtained compared to using the symmetric model Supplementary Equation 31. Defined in this way, the asymmetry scales with the waveguide noise bath and the Fano parameter q, which is independent of any other parameters. We find excellent

agreement between this model and the measured broad band noise spectra at high pump powers (see main text). It is important to point out that only the relevant bath occupancies n_m, n_r, $n_{b,\mathrm{wg}}$ as well as q (in the case of the highest drive powers) are actual fit parameters, while all other relevant parameters are extracted from - or verified in - independent (low drive power) measurements.

Relation to the displacement spectrum

In the weak coupling regime we can relate the single sided displacement spectrum $S_\mathrm{x}(\omega)$ using

$$\frac{S(\omega)}{\hbar\omega} = \frac{S_\mathrm{x}(\omega)}{x_\mathrm{zpf}^2}\frac{\kappa_e}{\kappa}\Gamma_+ \tag{35}$$

with the photon scattering rate $\Gamma_+ \approx 4G^2/\kappa$ for the optimal detuning $\Delta_{r,d} = \omega_m$, and the factor κ_e/κ taking into account the limited collection efficiency of photons leaving the cavity.

Supplementary Note 7: Low drive power limits

At low drive powers and sufficient shielding from room temperature Johnson noise, it is a very good approximation to set $n_{b,\mathrm{wg}} \to 0$. Eliminating the waveguide noise input allows for a significant simplification of the power spectrum

$$S(\omega) = \hbar\omega_d 10^{\mathcal{G}/10}\Big(1 + n_\mathrm{add} + \frac{4\kappa_\mathrm{e}(n_r\kappa(\gamma_{m,i}^2 + 4(\omega_m-\omega)^2) + 4n_{b,m}\gamma_{m,i}G^2)}{|4G^2 + (\kappa + 2i(\Delta_{r,d}-\omega))(\gamma_m + 2i(\omega_m - \omega))|^2}\Big), \tag{36}$$

with the mechanical noise bath $n_{b,m}$ related to the mechanical occupation n_m in Supplementary Equation 33. At low drive photon numbers we see no indication of TLS, pump phase noise, or waveguide heating of the cavity. The chosen attenuation and shielding of input and output microwave lines connecting the sample to room temperature Johnson noise limits the expected cavity occupation to $n_r \leq 0.01$. Under these assumptions, which are verified by our low power measurements (constant background noise, no cavity noise peak), we can simplify the power spectrum to the more standard form used in cavity electro- and optomechnics

$$S(\omega) = \hbar\omega_d 10^{\mathcal{G}/10}\Big(1 + n_\mathrm{add} + \frac{16 n_{b,m}\kappa_\mathrm{e}\gamma_{m,i}G^2}{|4G^2 + (\kappa + 2i(\Delta_{r,d}-\omega))(\gamma_m + 2i(\omega_m - \omega))|^2}\Big). \tag{37}$$

Without resonator occupation, Supplementary Equation 33 simplifies to

$$n_m = n_{b,m}\left(\frac{\gamma_{m,i}}{\kappa}\frac{4G^2+\kappa^2}{4G^2+\kappa\gamma_{m,i}}\right) \approx n_{b,m}\Big(\frac{1}{C+1}\Big), \tag{38}$$

where we introduced the cooperativity $C = 4G^2/(\kappa\gamma_{m,i})$ and assumed moderate coupling strength $4G^2 \ll \kappa^2$ in the last step.

Supplementary Note 8: Linear response limit - system calibration

For very small drive powers where $C \ll 1$ we can simplify the expected thermal noise spectrum further. Dropping terms associated with backaction allows to measure the displacement noise in a self-calibrated way. This compact model is particularly useful to back out g_0 and the system noise temperature with a minimal number of assumptions.

Starting with Supplementary Equation 37 we can make the replacement $n_{b,m} \approx n_m$ and drop the backaction term in the denominator. Both is valid for $C \to 0$. We then insert $G = \sqrt{n_d}g_0$ with the drive photon number defined in Supplementary Equation 7. We furthermore assume that the gain of the system is flat over the relevant detuning such that we can introduce the directly reflected pump power measured at the spectrum analyzer $P_\mathrm{r} = 10^{\mathcal{G}/10}P_\mathrm{out}$. The cavity output power is related to the cavity input power via Supplementary Equation 6

$$|S_{11}|^2 = \frac{P_\mathrm{out}}{P_\mathrm{in}} = \frac{4\Delta_{r,d}^2 + (\kappa - 2\kappa_e)^2}{4\Delta_{r,d}^2 + \kappa^2}. \tag{39}$$

Finally, we can write the measured noise spectrum, normalized by the measured reflected pump tone

$$\frac{S(\omega)}{P_{\mathrm{r}}} = \mathcal{O} + \frac{64 n_m \kappa_e^2 \gamma_{m,i} g_0^2}{(4\Delta_{r,d}^2 + (\kappa - 2\kappa_e)^2)(\kappa^2 + 4(\Delta_{r,d} - \omega)^2)(\gamma_{m,i}^2 + 4(\omega_m - \omega)^2}. \tag{40}$$

Only directly measurable system parameters and the temperature of the mechanical mode need to be known to extract g_0 without any further assumptions about the particular gain, attenuation or noise temperature of the chosen measurement setup. Knowing g_0, it is easy to accurately back out the input attenuation $\mathcal{A} = -66.3$ dB and drive photon number n_d (for example from an EIT measurement). Furthermore, from the measured offset

$$\mathcal{O} = (1 + n_{\mathrm{add}}) \frac{4\kappa_e}{n_d(4\Delta_{r,d}^2 + (\kappa - 2\kappa_e)^2)} \tag{41}$$

we conveniently infer the system noise temperature in units of photons $n_{\mathrm{add}} \approx 30$. The system gain $\mathcal{G} \approx 46$ dB is then easily determined from the not-normalized wide band background of the measured noise spectrum $S(\omega)$.

SUPPLEMENTARY REFERENCES

[1] Mohan, S., del Mar Hershenson, M., Boyd, S. & Lee, T. Simple accurate expressions for planar spiral inductances. *Solid-State Circuits, IEEE Journal of* **34**, 1419–1424 (1999).

[2] Kern, W. The evolution of silicon wafer cleaning technology. *J. Electrochem. Soc.* **137**, 1887–1892 (1990).

[3] Pitanti, A. *et al.* Strong opto-electro-mechanical coupling in a silicon photonic crystal cavity. *Opt. Express* **23**, 3196–3208 (2015).

[4] Yan, G.-Z., Chan, P. C., Hsing, I.-M., Sharma, R. K. & Sin, J. An improved TMAH Si-etching solution without attacking exposed aluminum. In *The Thirteenth Annual International Conference on Micro Electro Mechanical Systems, 2000. MEMS 2000.*, 562–567 (2000).

[5] Fujitsuka, N., Hamaguchi, K., Funabashi, H., Kawasaki, E. & Fukada, T. Aluminum protected silicon anisotropic etching technique using TMAH with an oxidizing agent and dissolved Si. *R&D Review of Toyota CRDL* **39**, 34–40 (2004).

[6] Walls, D. F. & Milburn, G. J. *Quantum Optics* (Springer, 1994).

[7] Fano, U. Effects of configuration interaction on intensities and phase shifts. *Phys. Rev.* **124**, 1866–1878 (1961).

[8] Geerlings, K. *et al.* Improving the quality factor of microwave compact resonators by optimizing their geometrical parameters. *Applied Physics Letters* **100**, 192601 (2012).

[9] Marquardt, F., Chen, J. P., Clerk, A. A. & Girvin, S. M. Quantum theory of cavity-assisted sideband cooling of mechanical motion. *Phys. Rev. Lett.* **99**, 093902 (2007).

[10] Dobrindt, J. M., Wilson-Rae, I. & Kippenberg, T. J. Parametric Normal-Mode Splitting in Cavity Optomechanics. *Phys. Rev. Lett.* **101**, 263602 (2008).

[11] Teufel, J. D. *et al.* Sideband cooling of micromechanical motion to the quantum ground state. *Nature* **475**, 359–363 (2011).

[12] Rocheleau, T. *et al.* Preparation and detection of a mechanical resonator near the ground state of motion. *Nature* **463**, 72–75 (2010).

[13] Fink, J. M. *et al.* Quantum-to-classical transition in cavity quantum electrodynamics. *Phys. Rev. Lett.* **105**, 163601 (2010).
